# A malignant choroid plexus tumor with heterologous differentiation and BAP1 deletion suggesting choroid plexus blastoma

**DOI:** 10.17879/freeneuropathology-2025-8899

**Published:** 2025-10-02

**Authors:** Arnault Tauziède-Espariat, Alice Métais, Léa Guerrini-Rousseau, Farah Sassi, Lauren Hasty, Raphaël Saffroy, Volodia Dangouloff-Ros, Kévin Beccaria, Euphrasie Servant, Pascale Varlet

**Affiliations:** 1 Department of Neuropathology, GHU Paris - Psychiatry and Neuroscience, Sainte-Anne Hospital, Paris, France; 2 Université de Paris, UMR S1266, INSERM, IMA-BRAIN, Institute of Psychiatry and Neurosciences of Paris, Paris, France; 3 Department of Children and Adolescents Oncology, Gustave Roussy, Université Paris-Saclay, Villejuif, France; 4 Molecular Predictors and New Targets in Oncology, INSERM U981, Gustave Roussy, Université Paris-Saclay, Villejuif, France; 5 Department of Biochemistry and Oncogenetic, Paul Brousse Hospital, Villejuif, France; 6 Department of pediatric Radiology, APHP, Hôpital Universitaire Necker Enfants Malades; INSERM U1299 and UMR 1163, Institut Imagine, Université Paris-Cité, Paris, France; 7 Department of Neurosurgery, Paris Hospitals Public Assistance, Hôpital Necker-University Paris Cité, Paris, France; 8 Department of pediatric Neurosurgery, APHP Hopital Universitaire Necker Enfants Malades, Université Paris-Cité, Paris, France

**Keywords:** Choroid plexus, Malignant, Heterologous components, BAP1, TP53

Pediatric intraventricular tumors are mainly represented by choroid plexus neoplasms. Among them, the choroid plexus carcinoma (CPC) represents the malignant form. This tumor type is characterized by frequent *TP53 *alterations, two methylation classes (adult and pediatric subtypes) (1,2), and can sometimes occur in the hereditary context of syndrome. Previously, our team reported a malignant plexus tumor presenting heterologous elements such as a pleomorphic spindle cell component and a polyphenotypic profile, which classified it as a choroid plexus tumor, subclass pediatric B by DNA-methylation profiling analysis (3). Herein, we present a morphologically similar choroid plexus tumor, this time harboring *BAP1 *and *TP53 *alterations.

A three-year-old girl, whose sister had Ewing sarcoma, presented with signs of intracranial hypertension. Magnetic resonance imaging (MRI) revealed the presence of a large tumor located in the posterior part of the left lateral ventricle. Cranial computed tomography (CT) scans revealed heterogeneous enhancement and intracranial calcifications (**Figure 1A–G**). CT scan of the chest, abdomen, and pelvis did not find other abnormalities. Germ cell markers were negative in the blood and in the cerebrospinal fluid. Gross total resection was achieved. Histopathologically, the tumor was pleomorphic with epithelial (arranged in solid sheets and papillary structures), mesenchymal (fascicles of spindle cells in a myxoid stroma and osteoid formation), and melanocytic elements (**Figure 2A–D**). There was no myogenic differentiation. The cellular density was high, with nuclear pleomorphism, necrosis, and frequent mitotic figures (> 20/2.3 mm^2^). Using immunohistochemistry, tumor cells were found to variably expressed cytokeratins AE1/AE3, CK18 (**Figure 2E**), GFAP, PS100, MITF, HMB45 (**Figure 2F**), desmin and smooth muscle actin. SMARCB1/INI1 (**Figure 2G**), BRG1/SMARCA4 (**Figure 2H**) and H3K27me3 expressions were maintained. There was no immunoreactivity for LIN28A, OLIG2, EMA, SALL4, myogenin, NUT, ETV4, BCOR or germinal markers (OCT3/4, PLAP, beta-HCG, and alpha-fetoprotein). There was no expression of p53, and Ki67/MIB1 was expressed in 70 % of cells (**Figure 2I**). Next-generation sequencing analyses of tumor cells evidenced a homozygous deletion of 3p21.1, including the *BAP1 *gene and a loss of function *TP53 *mutation (p.Y220C). RNA-sequencing analysis did not identify any fusion transcript. DNA-methylation profiling analysis classified the tumor as a CPC, pediatric subtype (calibrated scores > 0.9 in both classifiers DKFZ v12.8 and Bethesda NIH v2.0). Using t-Distributed Stochastic Neighbor Embedding analysis, the tumor clustered with a case previously reported (3), in close vicinity to CPC, pediatric subtype (**Figure 3A–C**). Whole exome sequencing analysis failed to reveal a *BAP1 *or *TP53 *germline alteration. Considering all these results, a complementary immunostaining of *BAP1* was performed and evidenced a loss of protein expression in tumor cells (**Figure 3B**). The patient received conventional and high-dose chemotherapy, followed by local radiation therapy. One year post treatment, a distant recurrence in the anterior right lateral ventricle was detected on imaging and confirmed by repeat resection. Histopathology was consistent with the initial tumor. At present, 21 months after the initial diagnosis, the patient is alive.

**Figure 1. Radiological features.**
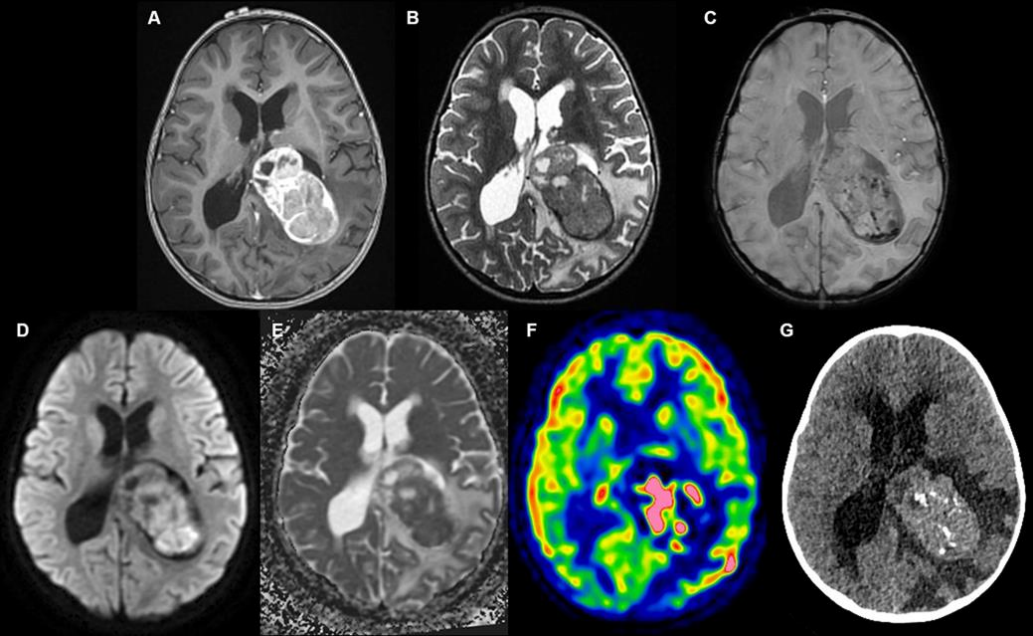
**A. **An intraventricular mass in contact with the choroid plexus showing heterogeneous enhancement after injection of gadolinium on T1-weighted computed tomography (CT) images. **B.** Intermediate T2 signal. **C **Calcifications and hemorrhagic changes on susceptibility weighted imaging. **D–E. **Diffusion restriction and low apparent diffusion coefficient. **F. **Elevated perfusion on arterial spin labeling perfusion imaging. **G. **CT scans showing intracranial calcifications.

**Figure 2. Histopathological features:**
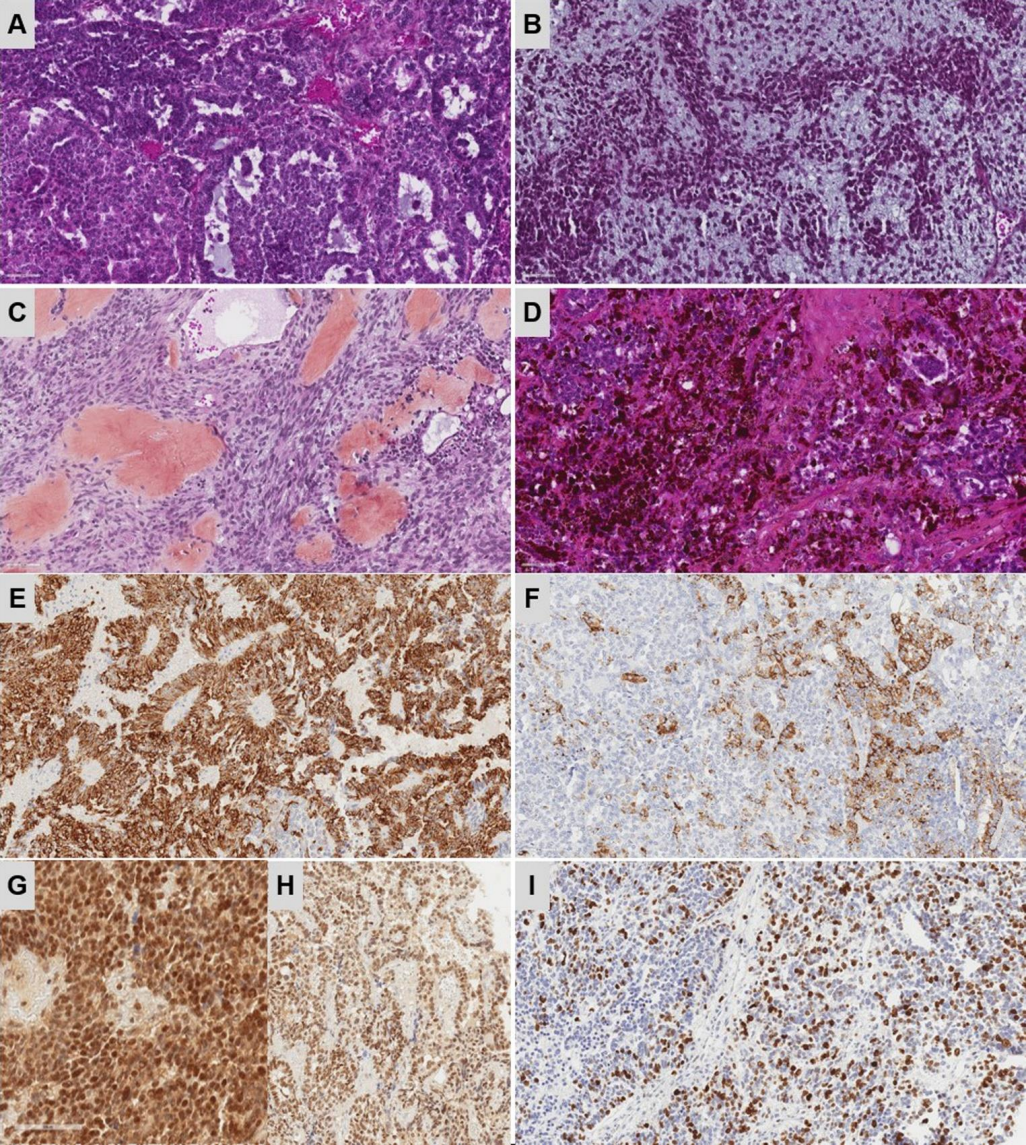
**A–D. **Histopathologically, the tumor was pleomorphous, and composed of an epithelial component with tubular structures, a mesenchymal component with spindle cells arranged in a myxoid stroma, or with an osteoid or melanin component (HPS, magnification x400). **E **Expression of CK18 (magnification x400) and HMB45 for a subset of tumor cells (**F. **magnification x400). Maintained expression of SMARCB1/INI1 and BRG1/SMARCA4 (**G–H. **magnification x400). High MIB1 labeling index (**I**, magnification x400).HPS: Hematoxylin Phloxin Saffron. Scale bars represent 60 μm. **Clicking into the picture will lead you to the full virtual slide** https://doi.org/10.57860/min_dts_000023

**Figure 3. Epigenetic features.**
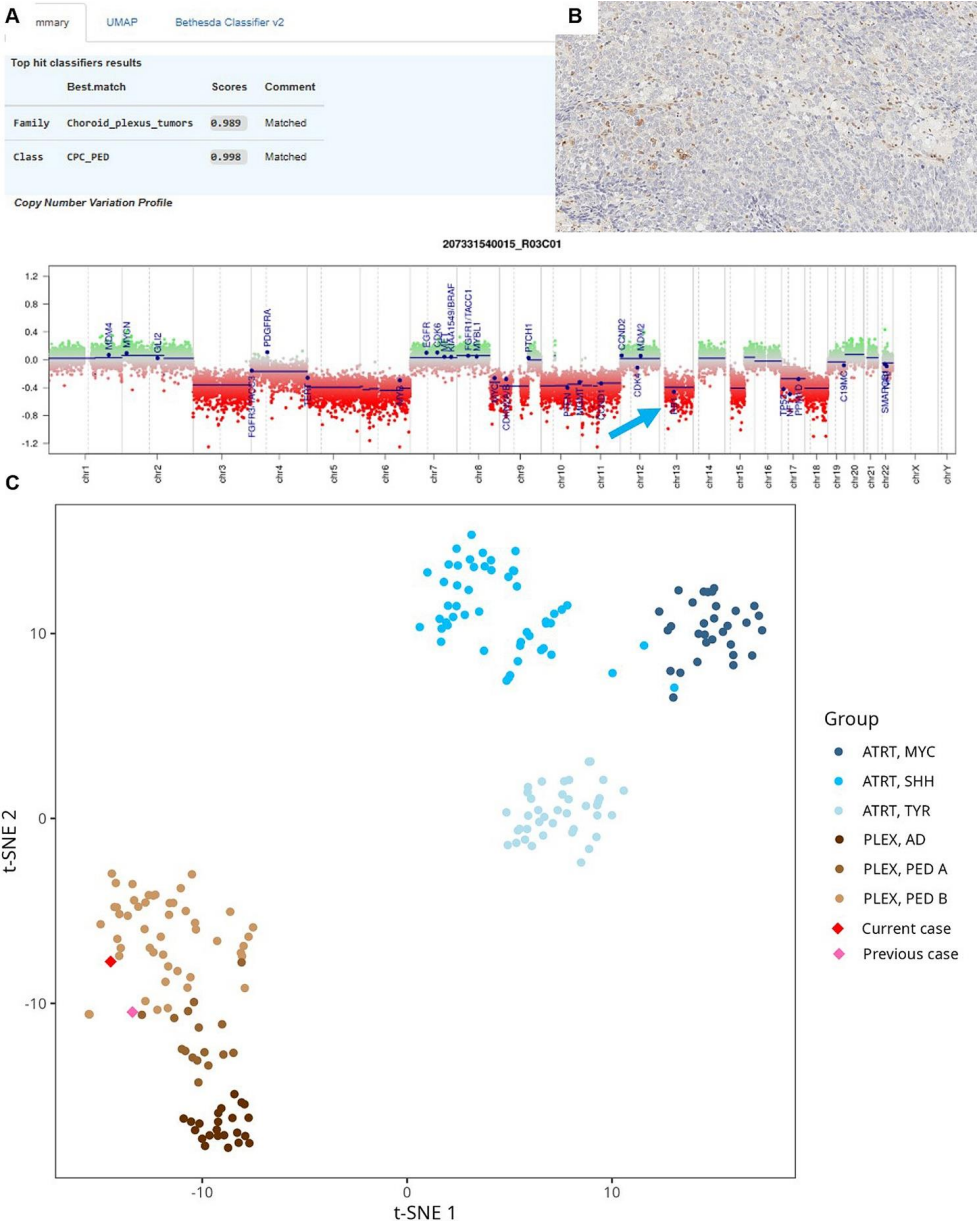
**A. **Copy number variation analysis of the tumor showed several aneuploidies without any amplification but a deletion of chromosome 13. **B. **Absence of protein *BAP1* immunolabeling in the tumor cells (magnification x400).** C.** t-Distributed Stochastic Neighbor Embedding analysis which included all DNA-methylation reference classes, showed that the current tumor clustered alongside the previously reported case of choroid plexus blastoma (3) and within the molecular subgroup "choroid plexus tumor, subclass paediatric B". The scale bar represents 60 μm.

In conclusion, we present the case of a pediatric malignant choroid plexus tumor with heterologous components and a *BAP1 *gene deletion. The current observation illustrates the diagnostic difficulties in pediatric tumors showing polyphenotypic histopathology. In the CNS, embryonal tumors, teratomas, and glioneuronal tumors can present heterologous differentiations. Rare observations of metaplasia have been reported in low-grade choroid plexus papillomas (7,8). The current intraventricular tumor contained a component resembling that of choroid plexus carcinoma. The differential diagnosis of a germ cell tumor was ruled out because of the negativity of germ cell markers, the absence of a mature teratomous component, and the epigenetic results classifying the tumor as a CPC. The DNA-methylation profiling was in line with this diagnosis, and the case was very similar to a previous observation in terms of clinics (age of onset, location), radiology, histopathology (presence of blastematous elements), and epigenetics (3). The current and the previous observations also share a familial context of tumors but without evidence of a germline alteration (particularly *TP53 *and *DICER1*). In contrast to the previous observation where immunohistochemical analysis retrospectively confirmed the maintained expression and no variant in *BAP1 *gene (3), the current case harboured a *BAP1 *deletion and a loss of protein expression, but no germline variant was evidenced. Moreover, no *BAP1 *deletion has been reported in CPC (1,2). Different tumor types have been identified as having somatic *BAP1 *alterations, and a subset belong to *BAP1-*tumor predisposition syndrome. In the CNS, meningiomas of various histologies (9) belong to this spectrum but no choroid plexus tumor has been reported to date. Here, there was no evidence for Li-Fraumeni-Syndrome and *BAP1*-tumor predisposition syndrome. Indeed, the tumor does not resemble other neoplasms with *BAP1 *deletions reported in extra-CNS locations, and the patient’s whole body imaging did not reveal any other tumor. While *BAP1* is generally not associated with Ewing sarcoma, ClinVar does report a single case with a likely pathogenic *BAP1* variant in the germline (see https://www.ncbi.nlm.nih.gov/clinvar/RCV000722041.1/). Our patient did not harbor any germline *BAP1* variants, suggesting that the occurrence of Ewing sarcoma in the sibling is coincidental.

We could like to point out that this case represents the second observation of a malignant choroid plexus tumor showing heterologous elements, thus highlighting the value of DNA-methylation profiling in the diagnosis of choroid plexus tumors with unusual histopathological features. Additional observations are needed to confirm if this neoplasm represents a novel tumor type (blastoma) or a subtype of choroid plexus carcinoma.

## Ethics approval

This study was approved by GHU Paris Psychiatry and Neuroscience, Sainte-Anne Hospital’s local ethics committee.

## Consent for publication

The patients signed informed consent forms before treatment began.

## Conflict of interest statement

The authors declare that they have no conflict of interest directly related to the topic of this article.
